# Barriers and enablers to implementing health education for HIV virologic suppression among adult people living with HIV-HBV coinfection in Northwest Ethiopia: a consolidated framework for implementation research guided qualitative study

**DOI:** 10.1186/s12981-026-00922-0

**Published:** 2026-07-06

**Authors:** Mequanente Dagnaw, Destaw Fetene Teshome, Tilahun Bizuayehu Demass, Abebaw Gebyehu Worku

**Affiliations:** 1 Department of Epidemiology and Biostatistics, Institute of Public Health, University of Gondar, Gondar, Ethiopia; 2 Department of Medical Biotechnology, Institute of Biotechnology, University of Gondar, Gondar, Ethiopia; 3 Department of Internal Medicine, College of Medicine and Health Sciences, University of Gondar, Gondar, Ethiopia; 4 Reproductive Health Department, Institute of Public Health, College of Medicine and Health Sciences, University of Gondar, Gondar, Ethiopia

**Keywords:** HIV-HBV coinfection, Patient-centered education, Virologic suppression, Ethiopia

## Abstract

**Background:**

Human immunodeficiency virus (HIV) remains a major public health challenge, particularly in sub-Saharan Africa, where achieving sustained HIV virologic suppression continues to be difficult despite expanded access to antiretroviral therapy (ART). This challenge is further complicated among people living with HIV-HBV coinfection due to increased disease complexity, treatment burden, and adherence-related barriers. Health education plays an important role in improving adherence and virologic outcomes; however, its implementation remains inconsistent in resource-limited settings. This study explored barriers and enablers to implementing health education for HIV virologic suppression among adults living with HIV-HBV coinfection in Northwest Ethiopia.

**Objective:**

This study aimed to explore the barriers and enablers influencing the implementation of health education for HIV virological suppression among adults living with HIV-HBV co-infection in North-West Ethiopia.

**Methods:**

A facility-based interpretive qualitative study was conducted from November 13, 2025, to January 12, 2026, at the University of Gondar Comprehensive Specialized Hospital and Felege Hiwot Comprehensive Specialized Hospital in Northwest Ethiopia. A total of 28 purposively selected participants, including adult people living with HIV-HBV coinfection, healthcare providers, and HIV program managers, participated in in-depth interviews (IDIs), key informant interviews (KIIs), and focus group discussions (FGDs). Data were audio-recorded, transcribed verbatim, translated into English, and analyzed using reflexive thematic analysis with a deductive approach guided by the Consolidated Framework for Implementation Research (CFIR) using MAXQDA Analytics Pro 2024. Trustworthiness was ensured through triangulation, member checking, peer debriefing, and maintaining an audit trail.

**Results:**

Ten barriers and eight enablers were identified across all CFIR domains. In addition to inaccurate information or graphics and transportation issues, the main reported hurdles included patient-level issues such as inadequate literacy, misconceptions about ART or viral load, anxiety about being seen attending sessions, and limited patient engagement in instructional design. Staff shortages, lack of commitment to counselling duties, a focus on ART refills rather than education, irregular or shortened sessions, lack of time, and the absence of a dedicated budget were all organizational and system-level barriers. Conversely, teamwork, clearly defined staff roles, the use of viral load monitoring tools, frequent review meetings, alignment with national HIV guidelines, patient motivation to achieve viral suppression, trust in provider advice, and improved adherence linked to education were all perceived enablers.

**Conclusion:**

Implementation of health education for HIV virological suppression among HIV-HBV co-infected patients is hindered by key barriers, particularly low patient literacy, misconceptions, stigma-related concerns, and systemic challenges such as staff shortages, limited time, and inadequate resources. However, strong teamwork, patient motivation, provider trust, and alignment with national guidelines serve as important enablers. Findings from this study informed the updating of the existing health education intervention to better address identified barriers and strengthen enabling factors. Testing the revised intervention for feasibility and acceptability is essential before wider implementation to improve adherence and achieve sustained HIV virological suppression in this population.

## Introduction

Human immunodeficiency virus (HIV) remains one of the most significant global public health challenges, particularly in sub-Saharan Africa, where the burden of infection, morbidity, and mortality remains disproportionately high [1]. Although the scale-up of antiretroviral therapy (ART) has substantially improved survival and reduced HIV-related morbidity, achieving and maintaining HIV virologic suppression remains a major challenge in routine HIV care [2]. Sustained virologic suppression is central to HIV treatment because it improves immune recovery, reduces opportunistic infections, enhances quality of life, and prevents onward transmission [2]. However, successful viral suppression depends not only on ART availability but also on optimal adherence, continuous follow-up, patient engagement, and effective health education interventions.

Hepatitis B virus (HBV) coinfection is common among people living with HIV (PLHIV) due to shared routes of transmission, including sexual contact, blood exposure, and vertical transmission [3]. Globally, HBV remains a major cause of chronic liver disease, and HIV-HBV coinfection presents substantial clinical and public health challenges [4]. HIV accelerates HBV replication and progression to liver fibrosis, cirrhosis, hepatocellular carcinoma, and liver-related mortality [4, 5]. Compared with individuals with HIV mono-infection, those with HIV-HBV coinfection often experience greater disease complexity, increased comorbidity burden, and poorer treatment outcomes.

Evidence suggests that achieving HIV virologic suppression may be more difficult among adults with HIV-HBV coinfection than among those with HIV alone [5, 6]. Multiple barriers contribute to this challenge, including increased pill burden, treatment fatigue, medication-related adverse effects, psychosocial stress, stigma, poor social support, and limited understanding of dual disease management [6, 7]. These barriers may adversely affect ART adherence, clinic attendance, retention in care, and long-term treatment success.

Health education is a cornerstone of comprehensive HIV care and plays a critical role in improving ART adherence, strengthening self-management, enhancing health literacy, and promoting sustained virologic suppression [8, 9]. Several strategies have been implemented to improve adherence and viral suppression, including structured counseling, peer support programs, reminder systems, community-based education, adherence clubs, and provider-led behavioral interventions [9]. These interventions aim to improve patients’ knowledge regarding ART benefits, viral load monitoring, adherence requirements, and the consequences of treatment interruption.

Despite these efforts, implementation of health education interventions remains inconsistent, particularly in resource-limited settings [10, 11]. Existing literature indicates that barriers occur at multiple levels. Patient-related barriers include stigma, low literacy, cultural beliefs, and economic hardship. Provider-related barriers include heavy workload, insufficient training, communication challenges, and burnout. Organizational and system-level barriers include staff shortages, limited educational materials, fragmented service delivery, and inadequate leadership support [11, 12]. Conversely, strong provider–patient relationships, institutional commitment, integrated care systems, and culturally tailored interventions have been identified as important enablers of successful implementation.

To better understand these complex implementation challenges, several implementation science frameworks have been applied in healthcare research. Common frameworks include the Reach, Effectiveness, Adoption, Implementation, and Maintenance (RE-AIM) framework, the Theoretical Domains Framework (TDF), and the Promoting Action on Research Implementation in Health Services (PARIHS) framework [13]. However, the Consolidated Framework for Implementation Research (CFIR) provides a more comprehensive and systematic approach by integrating multiple determinants of implementation across five domains: intervention characteristics, outer setting, inner setting, characteristics of individuals, and implementation processes [14, 15]. Because implementation of health education for HIV virologic suppression involves complex interactions among patients, providers, organizations, and health systems, CFIR was selected as the guiding framework for this study to systematically identify barriers and enablers influencing implementation.

Although substantial research has examined ART adherence and virologic outcomes among PLHIV, important evidence gaps remain regarding the implementation of health education interventions specifically for adults living with HIV-HBV coinfection, particularly in Ethiopia. Most previous studies have focused primarily on biomedical outcomes rather than implementation processes and contextual determinants influencing intervention success. Moreover, evidence from Northwest Ethiopia remains scarce despite the high burden of HIV and the growing need for integrated management of coinfected patients.

Understanding implementation barriers and enablers is essential for designing context-specific and sustainable interventions. Importantly, the findings of this study were intended not only to identify implementation challenges and enabling factors but also to inform the redesign and modification of the existing health education intervention guideline for adult people living with HIV-HBV coinfection. The revised intervention guideline is expected to strengthen patient-centered educational strategies and improve ART adherence and HIV virologic suppression. Therefore, this study aimed to explore the barriers and enablers to implementing health education for HIV virologic suppression among adult people living with HIV-HBV coinfection in Northwest Ethiopia using a CFIR-guided qualitative approach.

## Methods

### Consolidated Framework for Implementation Research (CFIR)

The Consolidated Framework for Implementation Research (CFIR) 2.0 guided the design, data collection, and analysis of this study. CFIR 2.0 provided a comprehensive framework for identifying and examining multilevel factors influencing the implementation of the Health Education on HIV virological suppression program, including intervention characteristics, inner setting, outer setting, individual characteristics, and implementation processes.

### Study design and period

A facility-based interpretive qualitative study was conducted to explore and understand the barriers and enablers influencing the implementation of health education for HIV virologic suppression among adult people living with HIV-HBV coinfection. In-depth interviews and focus group discussions were conducted with medical professionals and patients receiving antiretroviral therapy (ART). The study was conducted from November 13, 2025 to January 12, 2026 in several public health facilities in Northwest Ethiopia.

### Study setting

The study was conducted at two major referral hospitals in Northwest Ethiopia: Felege Hiwot Comprehensive Specialized Hospital (FHCSH), located in Bahir Dar City, and the University of Gondar Comprehensive Specialized Hospital (UoGCSH), located in Gondar City. These hospitals serve as major referral and teaching centers, providing specialized healthcare services to a large catchment population from the Amhara region and neighboring areas.

Both hospitals deliver comprehensive HIV prevention, care, and treatment services through dedicated ART clinics. These services include HIV testing and counseling, initiation and monitoring of antiretroviral therapy (ART), routine viral load monitoring, adherence counseling, management of opportunistic infections, and long-term follow-up care for people living with HIV (PLHIV). In addition, both facilities provide screening, diagnosis, and clinical management for HIV-hepatitis B virus (HIV-HBV) co-infected patients, including integrated care and treatment follow-up. The hospitals were selected because of their high patient load and well-established HIV care programs, making them suitable settings for the study [16, 17].

### Study population

The study population comprised three participant groups: HIV-HBV co-infected adult patients, healthcare providers, and HIV program managers or decision-makers. Although the intervention targeted HIV-HBV co-infected adults, additional stakeholder groups were included to obtain a comprehensive understanding of factors influencing implementation across multiple levels of the healthcare system.

HIV-HBV co-infected adults (aged 18 years or older) receiving ART services at the study hospitals were included because they were the primary recipients of the health education intervention and could provide firsthand experiences regarding its acceptability, relevance, and perceived impact.

Healthcare providers, including ART clinic nurses, physicians, adherence counsellors, pharmacists, and laboratory professionals, were included because they were directly involved in delivering HIV care and health education services and could identify practical barriers and enablers affecting intervention implementation.

Program managers and decision-makers, including HIV program coordinators, facility managers, and regional health officials, were included because of their roles in planning, supporting, and overseeing HIV services. Their perspectives helped identify organizational, resource, and policy-level factors influencing the adoption, implementation, and sustainability of the intervention.

### Inclusion and exclusion criteria

#### Inclusion criteria

Eligible participants were recruited according to the sequential phases of the study. First, persons aged 18 years or older living with HIV-HBV coinfection who were receiving antiretroviral therapy (ART) and had participated in or been exposed to the health education program on HIV virologic suppression were included. Second, healthcare professionals involved in providing ART and HIV care services were recruited. Third, HIV program coordinators, managers, supervisors, policymakers, and other key stakeholders involved in the planning, oversight, and implementation of HIV programs were included as key informants for key informant interviews (KIIs). To ensure informed and experience-based perspectives on program implementation, all participants were required to have at least six months of experience with, involvement in, or exposure to the health education program.

#### Exclusion criteria

Exclusion criteria were applied according to the sequential phases of the study. First, persons living with HIV-HBV coinfection who were seriously ill, cognitively impaired, unable to communicate effectively during data collection, or had not participated in or been exposed to the HIV virological suppression health education program were excluded. Patients with less than six months of exposure to the health education program were also excluded.

Second, healthcare professionals who were not actively involved in providing HIV care services during the study period, were on leave, or had less than six months of experience in HIV care service delivery were excluded.

Third, HIV program coordinators, managers, supervisors, policymakers, and other key stakeholders who were not directly involved in the planning, oversight, implementation, or monitoring of HIV programs, or who had less than six months of experience in their respective roles, were excluded from the study.

### Sample size, sampling procedures and data saturation

The sample size for this qualitative study was determined using the principle of thematic saturation rather than a predefined numerical target. Data collection and preliminary analysis were conducted concurrently to guide recruitment and assess emerging findings. Saturation was evaluated independently and through consensus discussions among the research team by reviewing interview transcripts, codes, and developing themes after each round of data collection. Recruitment continued until no new codes, themes, or substantive insights emerged across interviews and Consolidated Framework for Implementation Research (CFIR) domains, indicating achievement of both code saturation and meaning saturation. This ensured adequate depth and breadth of data to address the study objectives. A total of 28 participants were included in the final analysis.

Purposive sampling was used to recruit participants with rich and relevant experience related to the implementation of health education for HIV virologic suppression among adults living with HIV-HBV coinfection. Maximum variation sampling was employed to capture diverse perspectives and enhance the comprehensiveness of findings. Variation was sought based on age, sex, duration of antiretroviral therapy (ART), healthcare facility, and professional roles. Participants were selected based on their direct experience with HIV care, involvement in program implementation, or exposure to the health education intervention, and their ability to provide detailed information relevant to the study objectives.

### Description of the intervention

This study explored the implementation of routine health education activities provided to individuals living with HIV-HBV co-infection within ART services. These educational activities aimed to improve patients’ knowledge, motivation, and self-management skills related to HIV treatment and virological suppression. Common topics included the importance of adherence to antiretroviral therapy (ART), HIV-HBV co-infection and its implications for liver health, healthy lifestyle practices, safe sexual behavior, medication adherence, regular clinical follow-up, viral load monitoring, and timely reporting of treatment-related concerns.

Health education was delivered through individual counselling, group education sessions, and educational materials routinely available at the participating hospitals. Counselling was provided by healthcare professionals during regular clinic visits, while group sessions were conducted according to local service arrangements and resource availability. No standardized curriculum or implementation manual was in use across the study hospitals at the time of data collection; educational content and delivery were largely guided by routine clinical practice and provider experience. The findings of this study were subsequently used to inform the prospective development of a structured patient-centered health education manual for individuals with HIV-HBV co-infection.

### Data collection tools and procedures

Data were collected in three sequential phases using qualitative methods to explore barriers and enablers influencing the implementation of health education for HIV virologic suppression among adult people living with HIV-HBV coinfection in Northwest Ethiopia.

*Phase 1: In-Depth Interviews (IDIs)*

In-depth interviews were conducted with individuals living with HIV who were receiving antiretroviral therapy (ART). These interviews explored participants’ personal experiences, perceptions, and challenges related to the health education campaign and HIV care services.

For all three phases, semi-structured interview guides were developed based on the domains and constructs of the Consolidated Framework for Implementation Research (CFIR), ensuring a systematic exploration of intervention characteristics, outer setting, inner setting, characteristics of individuals, and implementation processes. The guides were reviewed by subject matter experts and pre-tested in a non-study setting to enhance clarity, relevance, and applicability.

*Phase 2: Key Informant Interviews (KIIs)*

Key informant interviews were conducted with HIV program managers, directors, and other key stakeholders involved in the planning, coordination, and implementation of HIV services. These interviews provided insights into program-level and organizational factors influencing implementation.

*Phase 3: Focus Group Discussions (FGDs)*

Focus group discussions were conducted with healthcare professionals involved in HIV care and treatment services. The FGDs facilitated exploration of shared experiences, perceptions, and contextual factors affecting the implementation of the health education campaign.

Experienced qualitative researchers conducted the data collection after receiving two days of training on qualitative interviewing techniques, research ethics, confidentiality, informed consent procedures, and application of the CFIR framework. Data collectors were closely supervised throughout the study to ensure adherence to study protocols and maintain data quality.

All IDIs, KIIs, and FGDs were conducted in participants’ preferred language, including Amharic, and were audio-recorded with participants’ consent. Recordings were transcribed verbatim and translated into English by bilingual professionals. To ensure translation accuracy and preserve the original meaning, a back-translation process was performed on a subset of transcripts.

### Data analysis

Data from in-depth interviews (IDIs), key informant interviews (KIIs), and focus group discussions (FGDs) were analyzed using reflexive thematic analysis with a deductive approach guided by the Consolidated Framework for Implementation Research (CFIR). All interviews and FGDs were audio-recorded, transcribed verbatim, and, where necessary, translated into English. The transcripts were then imported into MAXQDA Analytics Pro 2024 software to facilitate data management, organization, and coding.

The five CFIR domains; Intervention/Innovation Characteristics, Outer Setting, Inner Setting, Characteristics of Individuals, and Process were used to develop an initial deductive coding framework. Data were coded and interpreted within these predefined domains to identify patterns related to barriers and enablers influencing program implementation. Throughout the analysis, the research team engaged reflexively with the data, critically examining their assumptions, interpretations, and positionality to generate nuanced insights and deeper contextual understanding.

Coding and theme development were conducted iteratively across IDI, KII, and FGD transcripts, allowing themes to evolve through continuous engagement with the data. Rather than emphasizing coding consensus, regular analytical discussions among the research team were used to refine interpretations and strengthen the analytical depth of the study. Codes were subsequently organized into higher-level themes representing key implementation barriers and enablers, which were then grouped into broader thematic categories and mapped back to the relevant CFIR domains to maintain theoretical coherence. Triangulation was achieved through the use of multiple data sources (clients, program managers, and healthcare providers) and multiple data collection methods (IDIs, KIIs, and FGDs), thereby enhancing the credibility, trustworthiness, and rigor of the findings.

### Trustworthiness of the study

To ensure the trustworthiness and rigour of this qualitative study, the principles of credibility, transferability, dependability, and confirmability were applied throughout the research process. Credibility was enhanced through prolonged engagement with participants, triangulation of data sources, and member checking to validate the accuracy of interpretations. Transferability was supported by providing rich and detailed descriptions of the study context, participants, and research procedures, enabling readers to assess the applicability of the findings to similar settings. Dependability was ensured by maintaining a comprehensive audit trail documenting methodological decisions and analytical processes. Confirmability was strengthened through peer debriefing, ongoing reflexive practice to minimize researcher bias, and the inclusion of verbatim participant quotations to ensure that interpretations remained grounded in the data.

### Ethical considerations

Ethical clearance for this study was obtained from the Institutional Research Ethics Review Committee (IRERC) (Ref: College of Medicine and Health Sciences, and Specialized Hospital-University of Gondar-Institutional Research Ethics Review Committee/108/11/2025; dated 12 November 2025). Written informed consent was obtained from all participants prior to their inclusion in the study. Participant confidentiality was strictly maintained through the use of coded identifiers, and all data were securely stored with access limited to authorized personnel only. Interviews were conducted in a private setting to ensure privacy and comfort. Participation in the study was entirely voluntary, and participants were informed of their right to withdraw at any time without any consequences to their care or services. The study was conducted in accordance with the principles of the Declaration of Helsinki, as well as applicable national and institutional ethical guidelines.

## Results

### Participant’s characteristics

A total of 16 HIV-HBV co-infected patients receiving ART participated in the in-depth interviews. Most participants were male, and the majority were aged between 31 and 40 years. Most participants resided in urban areas. Educational attainment varied, with many participants having limited formal education, ranging from inability to read and write to primary-level education. Participants represented diverse occupational backgrounds, including government employees, NGO and private-sector employees, merchants, daily laborers, and others (Table 1).

**Table 1:** Socio-demographic characteristics of HIV-HBV co-infected patients participating in IDIs (*n* = 16)

Variable	Category	*n*
Sex	Male	11
	Female	5
Age (years)	18–30	6
	31–40	10
Residence	Urban	10
	Rural	6
Educational status	Cannot read and write	6
	No formal education	4
	Primary education	3
	Secondary education	1
	Higher education and above	2
Occupation	Government employee	3
	NGO employee	4
	Private organization employee	3
	Merchant/Trader	2
	Daily laborer	3
	Other	1

A total of 12 healthcare professionals and key informants participated in the study, comprising two HIV clinic physicians, two HIV clinic nurses, two ART adherence counsellors, two health educators, two ART clinic heads, and two hospital medical directors. Males constituted the majority of participants. Most participants had between six and ten years of professional experience, reflecting substantial involvement in HIV service delivery, program management, and implementation of HIV-related interventions (Table 2).

**Table 2:** Characteristics of healthcare professionals and key informants participating in FGDs and KIIs (*n* = 12)

Variable	Category	*n*
Participant Category	Healthcare professionals (FGDs)	8
	Key informants (KIIs)	4
Professional Role	HIV Clinic Physicians	2
	HIV Clinic Nurses	2
	ART Adherence Counsellors	2
	Health Educators	2
	ART Clinic Heads	2
	Hospital Medical Directors	2
Sex	Male	8
	Female	4
Work Experience (years)	1–5	6
	6–10	4
	11–15	2

### Barriers to health education intervention

The research identified several perceived barriers that hindered the successful implementation of the health education intervention for HIV virological suppression. These barriers were found in various CFIR categories, including the implementation process, individual characteristics, external and internal settings, and intervention features (Table 3).

**Table 3:** Participants’ perspectives on barriers to health education intervention by theme

Themes	Subthemes	Code
Intervention-Related	Complexity of the Education Package	Difficult for low-literacy patients (P 2 & 13)
	Design and Packaging Limitations	Unclear messages or visuals (P 18, 23 & 28)
	Resource Requirements	Requires time staff don’t have (P 10 & 11)
Patient-Related	Stigma and Fear of Disclosure	Fear of being seen attending sessions (P 20 & 26)
	Low Knowledge, Motivation, and Readiness	Misconceptions about ART or viral load (P 4 & 6)
	Socioeconomic Constraints	Transport barriers (P 22 & 24)
Health System	Human Resource Constraints	Staff shortages (P 2 & 9)
Provider-Related	Attitudes and Beliefs of Healthcare Workers	Low commitment to counselling tasks (P 6 & 26)
	Competing Clinical Priorities	Focus on ART refills over education (P1)
Implementation-Process	Poor Planning and Scheduling	Irregular scheduling of education sessions (P3 &15)
	Weak Execution and Fidelity	Sessions often shortened or skipped (P 10 & 17)
Policy and External Environment	Weak Policy Support	No dedicated budget for health education (P12)

#### Complexity of materials for low-literacy patients

Some patients struggle to comprehend written documents, medical terminology, directions, and counselling messages due to low literacy. One healthcare provider said that we must reiterate several times because *“patients who cannot read find concepts like viral load and suppression difficult” (*P2, ART ward nurse). This creates a barrier in a patient-centered health education intervention, particularly HIV/HBV education, because patients find it difficult to read or comprehend flipcharts, manuals, or appointment cards; they find it difficult to adhere to guidelines on viral load monitoring or medication adherence. They could be reluctant to ask questions out of shyness or embarrassment. Health professionals must spend more time explaining information since complex concepts (liver health, co-infection, viral suppression) are difficult to understand, which slows down sessions. Low literacy thus restricts the effectiveness of the educational intervention in terms of its reception, comprehension, and implementation. One of the patients said that *“health education that uses many words or written materials can be difficult for some of us to understand because we are not good readers”* (P13, HIV-HBV co-infected patients). Patients with low literacy skills found it difficult to comprehend basic concepts because health messages were viewed as being excessively technical or text-heavy.

#### Unclear messages or visuals

Patients may feel overloaded and less inclined to engage in the educational session if messages are presented in medical jargon or with intricate explanations. *“The messages contain clinical terminology*,* and I don’t always understand what they’re saying”* (P23, HIV-HBV co-infected patients). Some visual aids were said to be unclear or inadequately culturally appropriate, which reduced their ability to effectively convey important concepts about viral suppression and ART adherence. The other study participant also said, *“Sometimes the explanations and images are difficult to understand*,* leaving me confused as to what they signify*,* and there are too many words in the images. It is difficult for someone with poor reading skills like me to follow”* (P18, HIV-HBV co-infected patients). Patients, particularly those with limited literacy, may find it difficult to understand important concepts concerning ART, adherence, or viral load if the instructional materials (flip charts, posters, pictures, or messaging) are not straightforward, understandable, or culturally relevant. *“There are too many words in the images. It is difficult for someone with poor reading skills like me to follow”* (P28, HIV-HBV co-infected patients).

#### Requires time staff don’t have

When healthcare professionals believe that providing the health education intervention will take more time than they can reasonably devote, it is referred to as “*requires time staff don’t have*.” This dilemma becomes a significant obstacle since it impacts the intervention’s sustainability, consistency, and quality. We are required to provide counselling, documentation, and follow-ups. “*Finding time for further study under this pressure is challenging”* (P10, ART ward clinic head). Heavy workloads and large patient numbers limited the amount of time available for formal health education programs. *“We simply don’t have enough time during our hectic clinic hours to provide appropriate health education”* (P11, hospital director).

#### Stigma

Patients, particularly those with HIV or HIV-HBV co-infection, frequently fear that they will be stigmatized, criticized, or the subject of rumors if others witness them attending an education session. This anxiety lowers attendance and inhibits involvement. Some patients were deterred from taking part in group or facility-based teaching because of the stigma associated with HIV care. “*Patients were afraid of being spotted at the education sessions*,* which deterred them from attending because they were worried about stigma and accidentally disclosing their HIV status*” (P20, HIV-HBV co-infected patients). Education sessions are held in semi-open or open areas in many clinics. Patients may be reluctant to participate in group discussions or sit for counselling because they feel vulnerable. “*Uncomfortable conditions brought on by a lack of privacy and a fear of being seen decreased patient involvement with the intervention*” (P26, HIV-HBV patients). Patients are concerned that they may be observed entering or taking part in an HIV-related session by someone they know, such as neighbors, coworkers, or family. Anxiety and avoidance rise as a result.

#### Misconceptions about ART or viral load

Key statements in the intervention may be questioned, disregarded, or rejected by patients who have incorrect beliefs (e.g., ART is dangerous, viral load can be determined by symptoms, or herbal medicines can substitute ART). This lessens the educational impact. The community’s ongoing misinformation contributed to opposition or misunderstanding of the sessions’ objectives. Patients may not appreciate or completely engage in the education intervention if they think they already “know” about ART or viral load, even if their understanding is incorrect. *“Patients’ motivation to participate in the education sessions was reduced*,* and their comprehension of the intervention content was impeded by misconceptions regarding ART and viral load” (*P4, ART clinic nurse). Social and cultural factors might encourage misconceptions. Delivering the intervention is more difficult because it takes time, regular counselling, and trust to correct them. *“Participants found it difficult to accept accurate information due to deeply ingrained myths and false beliefs*,* which decreased the effectiveness of the health education program” (*P6, ART health educator).

#### Transport challenge

Some people find it exhausting to walk long distances or wait lengthy hours for transportation, especially those with comorbidities (such as HIV-HBV), which makes them less inclined to participate in educational programs. Long distances, limited transportation options, and associated expenses impeded regular attendance, especially for individuals residing in rural areas. *“I find it difficult to stay for the instruction because I am already exhausted from walking by the time I arrive at the clinic”* (P22, HIV-HBV co-infected patients). A large number of patients reside distant from the clinic. They are discouraged from attending planned health education seminars when they have to travel great distances, especially if they have to attend multiple times. Some patients may not be able to afford minibuses, motorcycles, or public transit. It becomes challenging for patients to attend sessions when they have to choose between transportation expenses and necessities. *“I occasionally miss the sessions because I don’t have enough money for transportation to the clinic”* (P24, HIV-HBV co-infected patients).

#### Staff shortages

Staff members are unable to devote enough time to properly conduct health education programs at hospitals or clinics when there are insufficient nurses, counsellors, or health officials. There was less time for patient education and conflicting professional duties as a result of scarce human resources. *“It’s difficult to find time to properly educate every patient because we don’t have enough staff”* (P9, ART ward clinic head). The few staff members have very little time for systematic health education because they must handle a variety of responsibilities, including clinical treatment, documentation, medication distribution, follow-up, and screening. Extended shifts and a heavy workload led to staff fatigue. Worn-out providers may rush or skip education sessions, thereby lowering quality and consistency. *“The workload wears us out*,* and by the time we complete clinical tasks*,* we are too exhausted to teach”* (P2, ART clinic nurse).

#### No continuous mentorship or follow-up

Patients frequently only receive instruction during clinic appointments; they are not regularly followed up with or mentored to reinforce what they have learned. Patients may struggle to apply knowledge, forget important instructions, or lose motivation to maintain adherence and behavior change if they don’t receive consistent advice, reminders, or supportive monitoring. Long-term HIV education outcomes like consistent medication use, viral load monitoring, and lifestyle modifications are impacted by this. *“They teach us once and then abandon us; eventually*,* we forget what to do when issues arise”* (P25, HIV-HBV patients). Due to a lack of opportunities for supportive supervision or refresher training, providers reported feeling less confident and consistent when delivering instruction. According to medical professionals, there is no formal mentorship program or planned follow-up to check on patients’ use of the knowledge they have gained during health education classes. Patients are not regularly assisted or monitored following counselling because of a severe workload, a shortage of personnel, and a lack of specialized follow-up protocols. Because health education needs to be reinforced over time, this lessens the intervention’s impact. *“After counselling*,* we simply move on to the next patient; there is no system to check if they understood or are practicing it.”* (P6, ART adherence counsellor).

#### Focus on ART refills over education

Antiretroviral treatment (ART) administration is frequently given precedence over patient education by healthcare professionals. Health education becomes secondary as a result of the high patient load, time constraints, and pressure to finish medicine refills on time. Consequently, there are fewer opportunities to reinforce adherence, viral suppression messaging, and self-management abilities since counselling and instruction sessions are hurried or neglected. *“We spend the majority of our time providing refills. There is not enough time left to adequately educate each patient”* (P1, ART ward clinician). Rapid ART distribution was often given precedence over interactive education by providers, particularly on busy clinic days.

#### Challenges in scheduling and delivery of health education sessions

Sessions of health education are not often offered. They might only take place during campaigns, when personnel are available, or at random times. Patients are not taught at regular intervals, which makes it challenging for them to remember important knowledge, purposefully attend sessions, or gain from consistent reinforcement. One of the participants said that *“they don’t always teach*,* but sometimes they do.”* We can never predict when schooling will take place.” (P15, HIV-HBV co-infected patients). Missed learning opportunities, less support for adherence, and uneven behavior change are the results of irregular scheduling. Inconsistent scheduling of sessions resulted in lost chances for patient involvement. One of the health care providers said *that “education doesn’t have a set time.” We only do it when we have free time.”* (P3, ART ward clinician).

Due to conflicting clinical responsibilities, a lack of staff, and a heavy patient load, health education programs are usually hurried, cut short, or omitted entirely at many healthcare facilities. Key lessons about drug adherence, virus suppression, and self-management are not properly conveyed when sessions are shortened. One of the participants said that *“because so many people are waiting*,* they teach very quickly*,* so we don’t get all the information”* (P17, HIV-HBV co-infected patients). Missed sessions deprive patients of crucial information and produce uneven learning experiences. Education exercises were often shortened or completely skipped *due to workload constraints or unforeseen clinic needs. One of the healthcare providers said that “we completely skip health education if the day is hectic.”* (P10, ART ward clinician).

#### Financial constraints

A dedicated budget for patient education initiatives is frequently absent from healthcare facilities. Without funding, clinics find it difficult to offer staff ongoing training, teaching resources (such as flip charts, manuals, and posters), or encouraging follow-up programs. Education is consequently underfunded, inconsistently provided, and viewed as an afterthought rather than a vital part of HIV care. *“Participants reported several financial challenges affecting health education delivery*,* including the absence of a dedicated budget for educational materials*,* staff training*,* and session implementation*,*”* said a healthcare provider (P12, hospital director). Funding was insufficient for facilities to support supplies, printing, or more staff time devoted to instruction.

### Enablers for health education intervention

The analysis found several enablers that made the health education intervention intended to improve HIV virological suppression easier to administer and more widely accepted. The CFIR domains of individual characteristics, intervention characteristics, inner setting, process, and outward setting encompassed these enablers (Table 4).

**Table 4:** Participants’ perspectives on enablers to health education intervention by theme

Themes	Subthemes	Code
Intervention-Related	Perceived Effectiveness of the Education	Encourages medication adherence (P 21 & P27)
Patient-Related	Strong Patient Motivation and Readiness	Desire to achieve viral suppression (P 16)
	Trust and Rapport with healthcare providers	Patients trust the provider’s advice (P19)
Health System	Supportive Work Environment	Teamwork among healthcare providers (P2)
Provider-Related	Positive Provider Attitudes	Belief in the effectiveness of health education (P3 &P7)
Implementation-Process	Good planning and organization	Defined roles among healthcare staff (P12)
	Monitoring and Continuous Improvement	Staff meetings used to review gaps and improve delivery (P3)
Policy and External Environment	Government and Partner Support	Alignment with national HIV guidelines (P 12)

#### Improved adherence through health education

Because it increases their capacity and incentive to regularly adhere to ART regimens, participants in HIV viral suppression programs frequently see promotion of medication adherence as a critical facilitator. Health education programs raise awareness, boost self-esteem, and dispel medication-related uncertainties, which facilitates and increases adherence. According to the participant, the health education seminars encouraged them to stick to their treatment plan by reiterating the significance of regular ART use. One participant said, *“I was reminded by the teaching seminars of the need to take my medications daily. I don’t miss my doses anymore”* (P27, HIV-HBV co-infected patients). Health education boosts knowledge, motivation, methods, and confidence, all of which support consistent ART use and eventually improve viral suppression outcomes, making it a perceived facilitator that promotes drug adherence. An ART follower in one of the hospitals said, *“I now understand why skipping my medication can spread the infection after the education sessions. That made me take my meds more carefully.”* (P21, HIV-HBV patients).

#### Patient motivation as a facilitator

Many patients have a strong desire to maintain a low or undetectable viral load. They actively participate in health education programs, ask questions, follow ART instructions, and adopt healthy habits because of their motivation. Patients are more willing to learn and put what they are taught into practice when they comprehend that viral suppression results in improved health, a longer life, and a lower chance of transmission. The efficacy of health education initiatives is enhanced by this motivation. One participant said, *“I pay attention to every message they teach us because I want to keep my viral load low”* (P16, HIV-HBV co-infected patients). Numerous patients conveyed a strong sense of personal drive to attain undetectable viral levels, which improved their response to adherence messages and counseling sessions.

#### Trust healthcare providers

Patients frequently have great faith in the skills and advice of healthcare professionals. Their propensity to attend health education courses, ask questions, heed counseling advice, and follow advised practices like taking ART consistently, abstaining from dangerous substances, and attending follow-up appointments is increased as a result of this trust. Patients are more likely to engage in educational interventions and apply what they learn when they feel their healthcare providers care about them. One participant said that *“I pay close attention to the nurses’ advice because I trust what they say”* (P19, HIV-HBV co-infected patients). High levels of confidence in healthcare professionals increased patients’ acceptance of the instructional material and increased their likelihood of adhering to recommendations about ART adherence, scheduling appointments, and lifestyle modifications.

#### Teamwork among healthcare providers

Because it allows for coordinated patient assistance, consistent messaging on ART adherence, collaborative problem-solving, and shared accountability for patient outcomes, teamwork among healthcare providers enhances successful health education. Patients receive clear, consistent, and ongoing health education when nurses, doctors, pharmacists, and counselors collaborate, which facilitates their understanding of the significance of viral load monitoring and ART adherence. One participant said, *“When we collaborate*,* everyone conveys the same message to the patient. This enables students to follow and take the education seriously”* (P2, ART clinic nurse). Clinicians, nurses, adherence counselors, and case managers collaborated well, according to providers. This collaboration made it easier to assign tasks and guarantee that education was integrated into daily care. The other participant said that *“working as a team makes health education easier because we share the responsibility that one person cannot do alone.”* (P2, ART clinic nurse).

#### Belief in the effectiveness of health education

Healthcare professionals are more inclined to commit time, give education regularly, and make sure patients comprehend the material when they firmly feel that health education improves ART adherence and viral suppression. Even in hectic clinical environments, this concept encourages dedication, zeal, and prioritizing of instructional tasks. One healthcare provider participant said that *“education is effective.” Patients seldom forget to take their medications when they know why they are taking ART”* (P7, ART adherence counselor). Health education was seen as a crucial part of HIV care by both patients and healthcare professionals. Participants said that education sessions strengthened adherence, enhanced self-management, and helped dispel misconceptions. One healthcare provider participant said, *“I make sure I give health education enough time because I know it changes behavior.”* (P3, ART ward clinic nurse).

#### Defined roles among healthcare staff

Because each cadre is aware of what to teach, when, and to whom, clearly defined duties among healthcare personnel help guarantee that health education is administered methodically and without being neglected. A seamless and well-coordinated information flow to patients is produced when duties are organized (for example, a doctor diagnoses, a nurse follows up, a counselor offers thorough instruction, and a pharmacist enforces adherence). One healthcare provider participant said that *“no patient misses health education when roles are clear.” " Everyone is aware of what to do”* (P12, Hospital director). Workflows were expedited, and task ownership increased when health education tasks were assigned. The staff was aware of who was in charge of counseling, monitoring, and reviewing issues related to adherence.

#### Continuous quality improvement processes

Programs for health education are strengthened by holding regular staff meetings to discuss issues, patient input, and service delivery gaps. Healthcare professionals can identify problems, clarify messaging, make changes, and divide up health education tasks during these meetings. The team thereby improves its coordination, consistency, and responsiveness to patient demands. One of the healthcare provider participants said that *“we talk about what is lacking in our patient education during meetings and work together to fix it”* (P3, ART ward clinic nurse). Frequent facility meetings served as important forums for talking about difficulties, exchanging information, and modifying the implementation strategy. During these sessions, providers reviewed patient progress, planned changes, and identified counseling gaps. Another health care provider participant said that *“our discussions enable us to improve the way we provide health education.”* Everyone gains knowledge from one another (P3, ART ward clinic nurse).

#### Alignment with national HIV guidelines

Participants are reassured that the information is correct, authoritative, and in line with national standards when it is in line with national HIV recommendations, which also boosts trust and the health education intervention’s perceived credibility. The health education program complied with national recommendations for viral load monitoring and HIV treatment. Providers reported that this alignment guaranteed consistency across facilities and gave them confidence in the content. Additionally, it facilitated the integration of the intervention into standard HIV services without necessitating significant adjustments to procedures. One participant said, *“I felt it was safe to follow because the instruction was in line with the national HIV standards. It clarified for me the significance of viral load monitoring and adherence for every patient.”* (P12, Hospital director).

## Discussion

This study explored barriers and enablers influencing implementation of a patient-centered health education intervention designed to improve HIV virological suppression among HIV-HBV co-infected adults. Using the Consolidated Framework for Implementation Research (CFIR), findings demonstrated that implementation was shaped by factors across all five CFIR domains: Intervention Characteristics, Outer Setting, Inner Setting, Characteristics of Individuals, and Process. The results highlight the complex interaction between patient-level, provider-level, organizational, and contextual factors that influence implementation effectiveness.

### Intervention characteristics

Within the CFIR domain of Intervention Characteristics, participants generally perceived the health education intervention as beneficial, relevant, and compatible with existing HIV care services. Healthcare providers reported that educational content aligned with national HIV treatment guidelines and viral load monitoring recommendations, enhancing its credibility and acceptability. Previous implementation studies have shown that interventions perceived as evidence-based and compatible with existing workflows are more likely to be adopted and sustained in routine practice [14, 18].

However, implementation challenges emerged regarding intervention adaptability. Providers reported difficulties tailoring educational content to patients with limited literacy and varying levels of HIV knowledge. Low literacy, misconceptions about ART, and misunderstandings regarding viral suppression reduced participants’ ability to fully engage with educational messages. Similar findings have been reported in HIV implementation research, where educational interventions require adaptation to local language, literacy level, and sociocultural context to achieve optimal effectiveness [1, 19].

### Outer setting

The Outer Setting domain revealed several patient-level and environmental factors affecting implementation. Patient motivation emerged as one of the strongest enablers. Many participants expressed a strong desire to achieve viral suppression, maintain good health, and avoid disease progression. This intrinsic motivation enhanced participation in health education sessions and encouraged adherence to ART. Previous studies have consistently identified patient motivation and perceived benefits of treatment as critical determinants of engagement in HIV care and sustained viral suppression [19, 20].

Trust in healthcare providers also facilitated implementation. Participants viewed healthcare workers as reliable sources of information and guidance, increasing acceptance of educational messages and adherence recommendations. Strong patient-provider relationships have been associated with improved retention in care, treatment adherence, and viral suppression outcomes across diverse HIV settings [21].

Conversely, stigma, fear of HIV status disclosure, and transportation challenges were major barriers. Many participants reported concerns about being identified while attending educational sessions or HIV clinics. Transportation costs and long travel distances further limited attendance. These findings are consistent with evidence from sub-Saharan Africa showing that stigma and structural barriers continue to impede access to HIV services and negatively affect treatment outcomes [1, 22].

### Inner setting

The Inner Setting domain highlighted the importance of organizational context in determining implementation success. Teamwork, role clarity, and collaborative work environments facilitated intervention delivery. Healthcare providers described effective coordination among clinicians, adherence counselors, case managers, and ART clinic staff. Clearly defined responsibilities reduced duplication of effort and enhanced continuity of patient support. Regular staff meetings also provided opportunities for performance review, problem-solving, and shared learning.

These findings are consistent with implementation science literature demonstrating that organizational readiness, effective communication networks, and collaborative cultures support successful implementation and sustainability of healthcare interventions [14, 23].

Despite these strengths, several organizational barriers were identified. Staff shortages, heavy workloads, lack of private counseling spaces, and competing clinical priorities limited providers’ ability to deliver comprehensive health education. ART refill services were frequently prioritized over counseling activities, resulting in shortened or missed educational sessions. Additionally, the absence of dedicated financial resources and weak integration with nutrition support services restricted implementation scope. Similar health-system constraints have been widely reported in resource-limited HIV care settings and remain significant barriers to implementation fidelity [19, 24].

### Characteristics of individuals

The Characteristics of Individuals domain captured factors related to both healthcare providers and patients. Positive provider attitudes toward the intervention supported implementation. Most healthcare workers recognized the value of health education in promoting adherence and improving viral suppression outcomes. Such positive beliefs about intervention effectiveness are known to facilitate implementation and long-term adoption [14].

However, some providers reported limited confidence in delivering counseling due to insufficient refresher training, mentorship, and supportive supervision. Lack of continuous professional development reduced motivation and may have contributed to variations in counseling quality. Previous implementation studies demonstrate that ongoing training, coaching, and supportive supervision improve provider competency, implementation fidelity, and patient outcomes [25, 26].

Patient knowledge and understanding also influenced implementation effectiveness. Misconceptions regarding ART, viral load monitoring, and HIV treatment outcomes created barriers to engagement with educational content. These findings suggest the need for simplified educational materials and culturally appropriate communication strategies tailored to diverse literacy levels.

### Process

The Process domain revealed several factors affecting implementation quality. Regular review meetings and performance monitoring mechanisms facilitated implementation by creating opportunities for reflection, feedback, and continuous improvement. Such activities strengthened accountability and enabled healthcare teams to address emerging challenges during implementation.

Nevertheless, important weaknesses in implementation processes were identified. Participants reported inconsistent scheduling of educational sessions, lack of standardized operating procedures, and inadequate follow-up mechanisms. These process gaps contributed to variability in intervention delivery across facilities. According to implementation science literature, successful implementation requires structured planning, ongoing monitoring, stakeholder engagement, and continuous evaluation to ensure fidelity and sustainability [14, 26].

The findings suggest that strengthening implementation of health education for HIV virological suppression requires interventions across multiple CFIR domains. At the intervention level, educational materials should be adapted for patients with low literacy and translated into local languages. At the outer-setting level, stigma reduction strategies and transportation support mechanisms should be considered. Within healthcare facilities, increased staffing, dedicated counseling spaces, and stronger integration with nutrition services may improve implementation capacity. Continuous mentorship, refresher training, and supportive supervision are needed to enhance provider confidence and competency. Finally, implementation processes should be strengthened through standardized protocols, routine monitoring systems, and regular feedback mechanisms.

## Limitations of the study

Several limitations should be considered when interpreting the findings of this study. Social desirability bias may have occurred, particularly among healthcare professionals, who may have provided favorable responses regarding their practices or health facilities. Because this qualitative implementation study was conducted in two referral hospitals in northwest Ethiopia, the findings may not be fully generalizable to other HIV care settings or regions. Recall bias may also have influenced participants’ accounts, as some individuals may have had difficulty accurately remembering previous experiences with health education interventions or virological monitoring. In addition, although efforts were made to ensure accurate translation and transcription, some nuances may have been lost during translation from local languages into English. Finally, the use of purposive sampling may have introduced selection bias, as individuals who were more engaged with HIV care services may have been more willing to participate, potentially underrepresenting the perspectives of less engaged or marginalized patients.

## Conclusion

Using the Consolidated Framework for Implementation Research (CFIR), this study identified multiple factors influencing the implementation of health education for HIV virological suppression among HIV-HBV co-infected patients. Key enablers included strong patient motivation, trust in healthcare providers, effective teamwork, clearly defined staff roles, and alignment of the intervention with national HIV guidelines. In contrast, implementation was hindered by patient-level barriers such as low literacy, stigma, misconceptions about HIV treatment, and transportation challenges, as well as health system constraints including staff shortages, limited time, inadequate infrastructure, insufficient resources, and weaknesses in implementation processes.

These findings demonstrate that successful implementation depends on addressing interconnected factors across the CFIR domains, including intervention characteristics, outer setting, inner setting, characteristics of individuals, and implementation processes. Based on these findings, the existing health education intervention was updated and refined to better address identified barriers and strengthen enabling factors. The revised intervention incorporates context-specific, patient-centered educational strategies tailored to the needs of adults living with HIV-HBV coinfection. Strengthening organizational capacity, enhancing provider support through training and mentorship, adapting educational materials to patients’ literacy levels, and addressing stigma and access-related barriers may improve implementation fidelity and patient engagement. The updated intervention will subsequently be tested to assess its feasibility, acceptability, and potential effectiveness in improving adherence to antiretroviral therapy and achieving sustained HIV virological suppression. Policymakers, program managers, and researchers should prioritize these strategies to support sustainable implementation and improved treatment outcomes.

## Data Availability

No datasets were generated or analysed during the current study.

## Abbreviations

AASLD: American Association for the Study of Liver Disease
ADEs: Adverse Drug Events
AIDS: Acquired Immune Deficiency Syndrome
ALT: Alanine Aminotransferase
ART: Antiretroviral Therapy
CHIV: Chronic Hepatitis B
DAAs: Direct-Acting Antivirals
ETV: Entecavir
FGD: Focus Group Discussion
HAART: Highly Active Antiretroviral Therapy
HIVeAb: Hepatitis B (e) Antibody
HIVeAg: Hepatitis B e-Antigen
HIVsAg: Hepatitis B surface Antigen
HCC: Hepatocellular Carcinoma
HCPs: Health Care Providers
HIV: Human Immunodeficiency Virus
HRQoL: Health-related quality of life
IDI: In-Depth Interview
IRB: Institutional Review Board
IV: Intra Veinous
KIIs: Key Informant
LMICs: Low-and Middle-Income Countries
MAXQDA: MAX Qualitative Data Analysis
MCS: Mental Component Summary
MeSH: Medical Subject Headings
MH: Mental Health
MOPs: Manual of Operating Procedures
NAs: Nucleotide Analogs
NUCs: Nucleotide analogs
OPD: Out Patient Department
PCPs: Primary Care Providers
PCS: Physical Component Summary
IFN-a: Interferon-alpha
PF: Physical Functioning
PLHIV: People Living with HIV
SF: Social Functioning
SSA: Sub-Saharan Africa
SVR: Sustain Virological Response
TDF: Tenofovir disoproxil fumarate
WHO: World Health Organization
